# Healthcare use among people using methamphetamine in Winnipeg, Canada

**DOI:** 10.23889/ijpds.v9i5.2566

**Published:** 2024-09-18

**Authors:** Jennifer Enns, Joykrishna Sarkar, Nathan Nickel, Amy Freier, Carolyn Shimmin, Ogai Sherzoi, Aaron Goss, Chelsey McDougall

**Affiliations:** 1 Manitoba Centre for Health Policy, University of Manitoba; 2 George and Fay Yee Centre for Healthcare Innovation, Shared Health Manitoba

## Objective

We examined healthcare use among people using methamphetamine who were connected to a Community Health Centre (CHC) in Winnipeg, Canada. CHCs are primary care clinics designed to provide person-centred, holistic care to people who may be impacted by discrimination and/or oppression.

## Approach

From population-based administrative health data, we identified people with a record of a methamphetamine-related healthcare system encounter from 2013-2020 (cases) and created a comparison group matched on age, sex, and postal code but with no recorded history of using methamphetamine (controls). We narrowed this population to those who visited a CHC at least once. Then, using negative binomial regression models adjusted for sociodemographic characteristics, we produced rate ratios and 95% confidence intervals for primary care visits, CHC visits, emergency service contacts, emergency department (ED) visits, and hospitalizations in the 5 years after the first recorded methamphetamine use.

## Approach

Adjusted rate ratios showed that healthcare use among CHC-connected people using methamphetamine was higher than among CHC-connected people not using methamphetamine: primary care 1.15 (95% CI 1.07-1.23); CHC 1.17 (95% CI 1.01-1.37); emergency services 6.19 (95% CI 5.46-7.02); ED 3.19 (95% CI 2.92-3.48); hospitalization 2.77 (95% CI 2.46-3.10).

## Conclusion & Implications

Being connected to a CHC may have facilitated access to other health and social services, but there is still unmet need for high quality, holistic care among people using methamphetamine. This research informs our team’s ongoing efforts to address mental health and addictions concerns across sectors and to promote anti-discriminatory approaches to addictions care in the healthcare system.

